# My Neighbor: Children’s Perception of Agency in Interaction with an Imaginary Agent

**DOI:** 10.1371/journal.pone.0044463

**Published:** 2012-09-07

**Authors:** Yusuke Moriguchi, Ikuko Shinohara

**Affiliations:** 1 Department of School Education, Joetsu University of Education, Joetsu, Japan; 2 Precursory Research for Embryonic Science and Technology, Japan Science and Technology Agency, Tokyo, Japan; 3 Department of Psychology, Aichi Shukutoku University, Nagakute, Japan; George Mason University/Krasnow Institute for Advanced Study, United States of America

## Abstract

Children may treat an invisible entity as a live and thinking entity, known as an imaginary companion (IC). Some researchers suggest that this is simply pretend play, but it is possible that children experience agency in their interactions with ICs. Given the literature on cognitive science and social brain research, we hypothesize that young children may have an agent-perception system that responds to an invisible agent by which they may experience realistic agency in their interactions with ICs. In this study, children were introduced to an invisible agent and an invisible stone. However, they assigned biological and psychological properties to the agent but not the stone. The tendency of assigning such properties was stronger in children with ICs than in those without ICs. These results contribute to our understanding of cognitive and neural development in typical and atypical children.

## Introduction

Piaget [1] proposed that children attribute biological (e.g., being alive) and psychological (e.g., having knowledge) properties to inanimate objects such as clouds, which is known as children’s animism. This finding was questioned by empirical evidence showing that preschool children can differentiate between animate and inanimate objects and that they do not believe that inanimate objects (e.g., rocks) grow, walk, and remember [2], [3].

Yet, children often treat objects or invisible entities as living and thinking beings, as seen in movies such as “My Neighbor Totoro.” These imaginary agents, or imaginary companions (IC), also participate in activities with children [4], [5]. Two types of ICs are generally observed: a stuffed animal and an invisible friend. The phenomenon of having an IC is commonly observed in young children with approximately half interacting with the ICs as they would a real friend [6], [7]. Researchers suggest that having an IC is just simply pretend play. Children are clear about the fantasy status of ICs, and discriminate between them and real friends [5], [8]. This implies that children’s animistic behaviors with ICs are more superficial than real.

However, even though children know that ICs do not exist, they may still experience reality in their interactions with the ICs. ICs were originally defined as “an invisible character … having an air of reality for the child…” [4]. Indeed, research indicates that mental imagery can reflect a certain reality for children [9], [10], [11]. Thus, it is possible that children may, to some extent, attribute biological and psychological properties to an imaginary agent. This idea is also supported by the literature related to the cognitive science of religion and social brain research. Cognitive scientists suggest that humans have a bias for detecting human-like agency and the agent detector may sometimes respond to a postulated agent who has unusual characteristics such as invisibility [12], [13]. The mechanism may be functional during childhood, when children attribute psychological properties to gods [14]. Given that children with ICs may have better socio-cognitive skills than those without IC [7], [15], it is possible such children with ICs are more likely to possess overactive agent detectors.

Recent social brain research has identified a brain network, considered to have a specific role in social cognition. It involves the posterior superior temporal sulcus (pSTS), temporo-parietal junction (TPJ), and the medial prefrontal cortex (MPFC) [16], [17]. The pSTS processes another person’s eye movement and biological motion (biological property) [18], while the TPJ plays an important role in considering others’ perspectives. The MPFC exhibits significant activities during mentalization (psychological property) [19]. The brain network is functional during early childhood [20]. Importantly, the pSTS has shown significant activation during both imaging and viewing biological motion [21], [22]. Similarly, the MPFC is active when participants imagine objects as animated ones [22].

**Figure 1:**
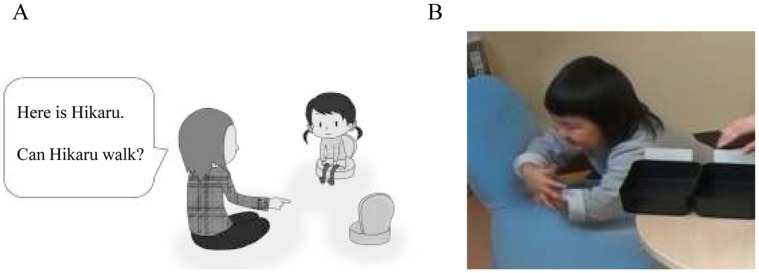
Experimental situation in the first study. (A) Children were introduced to an invisible agent named Hikaru and asked a sequence of questions about the properties of the agent. (B) A participant hugging Hikaru.

Given these considerations, it is possible that children may have an agent perception system that responds to imaginary and invisible agents by which they attribute biological and psychological properties to ICs. Specifically, this tendency would be observed in children with ICs but not in those without IC. In order to compare children with and without ICs, we devised an artificial IC paradigm based on fantasy/reality distinction research [11]. Children were introduced to an invisible agent named “Hikaru” [23] by a researcher (Figure 1) and then given a sequence of questions about the biological (e.g., “Does X walk?”), psychological (e.g., “Can X feel happy?”), and perceptual (e.g., “Can X see things?”) properties of the agent [24]. If our hypothesis is correct, children with ICs may attribute biological and psychological properties to Hikaru, while those without ICs may not.

## Results

### Study 1: A Human, a Ball, an Imaginary Agent

**Figure 2:**
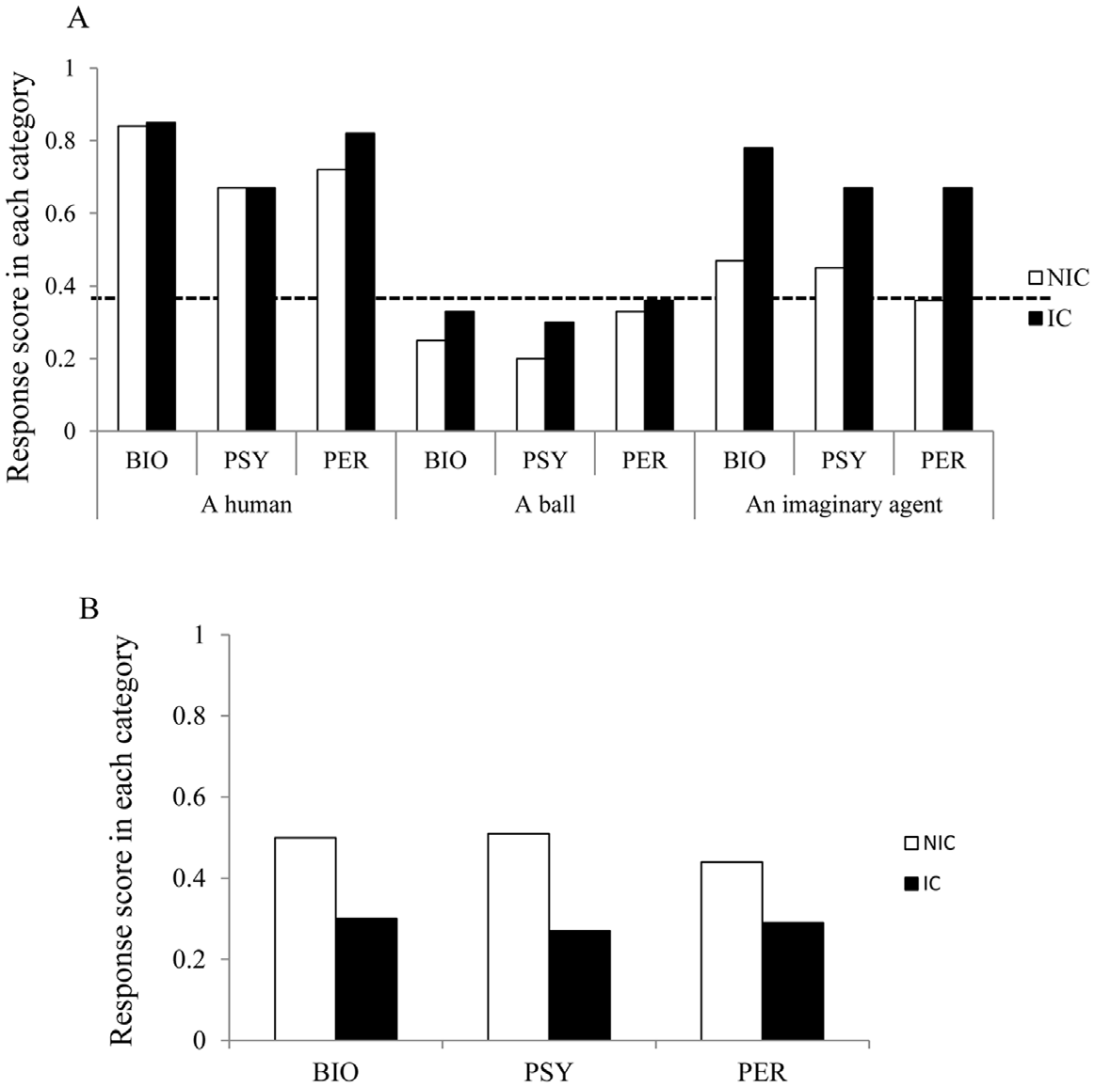
Results of the experiments. (A) Study 1. The response score was the proportion of “yes” responses (range 0–1.0). Score 0 means that children did not regard the item as an agent, whereas score 1 means that children attributed the item with biological and psychological properties. BIO = Biological, PSY = Psychological, PER = Perceptual. (B) Study 2.

Children with ICs (IC group, N = 26) and without ICs (NIC group, N = 24) participated in the first study. No significant differences were noted in the chronological and verbal ages between the groups (Table 1). As seen in Figure 2A, their responses to the questions were scored for the proportion of “yes” responses based on the question properties (biological, psychological, perceptual) and the items (human, ball, Hikaru) (range 0–1.0). First, we examined whether the response scores were different from the chance level (0.5). Children in both groups attributed each property to the human significantly above the chance level (P<.05), whereas their response scores for the ball in each category were significantly below the level (P<.05). However, the groups scored differently when asked about the imaginary agent. The response scores of the NIC children were at the chance level in each category (P>.10), whereas those of the IC children were all significantly above the level (P<.05).

**Table 1:** Participant profiles.

	Chronological Age	Verbal Age	Girl	First-born
Study 1				
NIC (N = 24)	52.1 (11.5)	55.8 (14.5)	33%	29%
IC (N = 26)	55.6 (14.3)	58.4 (13.6)	68%	72%
	n.s.	n.s.	*	*
Study 2				
NIC (N = 17)	52.0 (10.6)	51.5 (13.3)	41%	29%
IC (N = 17)	54.2 (13.8)	57.6 (14.8)	77%	76%
	n.s.	n.s.	*	*

Note. *P<.05.

Next, we compared the response scores of the IC children with those of the NIC children in each category by conducting an ANOVA test comparing group (IC/NIC) × item (human, ball, imaginary agent) on each property. We found a significant interaction between group and item related to biological properties [F (2, 86) = 3.414, P<.04], and a marginally significant interaction with perceptual properties [F (2, 86) = 2.714, P<.08]. A simple main effect analysis revealed that the IC children showed significantly higher response scores of the imaginary agent than did the NIC children (P<.05) in both biological and perceptual properties. However, no significant differences were noted in the scores of the human and ball items between the groups (P>.10). Furthermore, we found no significant interactions between group and item in the psychological properties [F (2, 86) = 1.878, P>.10].

### Study 2: An Imaginary Stone

Since IC children may attribute biological and psychological properties to any invisible item, we conducted a second study in which IC (N = 17) and NIC (N = 17) children were given questions about an imaginary (invisible) stone. If the IC children attribute animacy and agency to any invisible item, then they should respond with “yes” to questions about the imaginary stone. After being asked the same questions as in the first experiment, the results revealed that the response scores of both groups were never significantly above the chance level. Furthermore, we found no significant differences in the response scores in each category between the two groups (*P*>.10) (Figure 2B). Thus, we refute the interpretation that children with ICs respond to any imaginary item in the same manner.

### Study 3: Spontaneous References to Imaginary Items

Finally, we examined whether children spontaneously referred to the biological and psychological properties of imaginary items. In this case, we created a situation where a child may spontaneously talk about the imaginary agent and the stone. Regarding the imaginary agent, the researcher told the children that Hikaru had left the room, and regarding the imaginary stone, that she had put the stone away.

Six of the 26 IC children talked about the biological or psychological properties of Hikaru and interacted with him (Figure 1B; Table 2; Movie S1), but the NIC children never exhibited such behaviors. We conducted a Fisher’s exact test and found that the IC children produced more spontaneous comments about Hikaru than did the NIC children (*P*<.05). Furthermore, none of the children talked about the imaginary stone.

**Table 2:** Examples of spontaneous references to an imaginary agent.

(After Hikaru “left” the room)
E1 & E2: Will he come back?
C: He may be pooping.
E1 & 2: (laughing)
C: Because he is late.
E1: He may be pooping.
E1: The bathroom is far. Please wait for a moment.
E2: OK, let’s see…
E1: While waiting…
C: (Spontaneously looking at the door) Hikaru came back!
E1: Did he come back?
C: He came back.
E1: I’ll ask Hikaru something. (to Hikaru) Where is the toy?
C: (Hikaru) said here.

Note: C = Child, E1 = The first experimenter, E2 = The second experimenter.

## Discussion

These studies reveal that young children may regard an imaginary agent as possessing biological and psychological states. Specifically, children with an IC exhibit such attributions but NIC children did not show such attributions. Thus, IC children may be different from NIC children in terms of their agent perception system, perhaps subserved by the social brain network. Presumably, their agent perception system might respond to both real humans and imaginary entities. It should be noted that the results cannot be explained by children’s general fantasy orientation since research shows that children with high fantasy orientation generally make clear distinctions between fantasy and reality [25]. It is unlikely that children with a higher fantasy orientation would assign psychological and biological attributions to fantasy entities.

Our conclusion that children truly experience realistic agency is supported by further analysis. Specifically, we examined whether their responses were reliably discriminated from pretending on the basis of research on scale error [26]. Children in the first experiment were independently coded in order to identify whether they were pretending or being serious in their responses. One child in the NIC group was coded as pretending, but the rest of the children in both groups were coded as seriously responding to the questions. These results rejected the hypothesis that the children pretended to attribute agency to imaginary agents.

Piaget [1] suggested that children were animistic as they attributed psychological and biological properties to inanimate entities, which was not supported by the recent empirical evidence [3]. The present study showed that young children *really* attributed psychological and biological properties to an imaginary agent. The results seem to be consistent with Piaget’s ideas as children attributed such properties to non-human entity. Nevertheless, in this study, an imaginary agent was introduced by an experimenter as “an agent”. Thus, children attributed psychological and biological status to an agent, which may not accord with an idea of animism in a strict sense.

Finally, the results contribute to our understanding as to why some children with developmental disorders have ICs but others do not. For example, research reports that children with Down syndrome tend to converse with ICs [27] and appear to have relatively good sensitivity to social stimuli compared to those with similar levels of cognitive delay [28]. Furthermore, autistic children have a lower level of creative and imaginative ability, and they rarely have ICs [29]. It is well known that autistic children may have deficits in the brain network processing of social and emotional stimuli, such as in the areas of the pSTS and MPFC [18]. Although still unclear, our agency perception hypothesis may explain why children with Down syndrome, but not those with autism, tend to have ICs.

## Materials and Methods

### Study 1: A Human, a Ball, an Imaginary Agent

#### Ethics statement

Participants were recruited from a registry of families maintained in the Child Development Lab at Joetsu University of Education. Written informed consent was obtained from the parents of children prior to their involvement in the study. The study was conducted in accordance with the principles of the Declaration of Helsinki and the study design was approved by the ethics review board at the Joetsu University of Education.

The parents of the child in Figure 1B and Movie S1 have given written informed consent, as outlined in the PLoS consent form, to publication of their photograph and movie.

#### Participants

Participants included fifty children (age range: 30–79 months, mean ± SD: 53.9±12.9). Of these 23 were boys and 27 girls. First-born children constituted 72% of the sample, of which 14 children had no siblings. All participants were from middle-class backgrounds. Parents provided written consent after being informed verbally of the study’s purpose. The experiments were approved by the local ethics committee.

Children were divided into the imaginary companion (IC) group and the no imaginary companion (NIC) group according to their responses during an interview, which used a modified version of Taylor, Cartwright, and Carlson [30]. Details of the interview are reported below. Twenty-six children were classified as the IC group (age range: 30–79 months, mean ± SD: 55.6±14.3), of whom eight were males and 18 females. The remaining 24 children were classified as the NIC group (age range: 35–76 months, mean ± SD: 52.1±11.5). These included 15 males and nine females. No significant differences were demonstrated between the ages of the two groups. In addition, we measured children’s verbal age by using the Japanese version of the Picture Vocabulary Test that was standardized and revised by Ueno, Nagoshi, and Konuki [31]. The mean verbal age was 58.4 months (SD = 13.6) in the IC group and 55.8 months (SD = 14.5) in the NIC group. No significant differences were found in the ages between the two groups.

#### Procedure

After playing with each child for several minutes, the researcher asked the child about an IC (“I’m going to ask you some questions about your friends. Some friends are real, like the kids who go to your school and the ones with whom you play. And some friends are imaginary. They might be invisible, or they might be a puppet. Do you have an imaginary friend, or have you ever had one?”) If the child answered “yes,” then the researcher asked the child questions about the imaginary friend (e.g., gender, age, physical appearance).

We also conducted the imaginary companion interview separately with the child’s parent. To avoid confusion, we told some stories about an invisible friend and a personalized object. We explained that children of this age were often secretive about their imaginary companions and it was common for parents to be unaware of such friends. Mothers were then asked if they were aware of their children engaging in imaginary companion play currently or at a previous time. If they responded yes, the rest of the imaginary companion interview was conducted. Children were considered to have an imaginary companion if either the children or their parents indicated the presence of an imaginary companion currently or in the past. Mothers were also asked about the children’s reports of imaginary companions to clarify whether the children had mistakenly identified a real friend. Such incorrect reports by children were not included as evidence of an imaginary companion.

In the IC group, five children had invisible friends and 21 had personalized objects. Examples of ICs are presented in Table 3. Eleven children (52%) in the IC group had only one IC, while the remaining children had two or more ICs. Children’s invisible friends consisted of people (all children) and dogs (one child), with most (95%) of the children’s personalized objects being animal puppets (dogs, penguins, bears, and so on). Twenty-one children currently had ICs, and five children had stopped playing with their ICs prior to testing.

**Table 3:** Descriptions of Imaginary Companions.

Name	
Invisible friends (N = 8^a^)	
Umechan	An invisible girl who taught the child everything
Akari	A small person named after a real friend
Stuffed animals (N = 35^b^)	
Kumachan	A male bear who was big and play with the child anytime
Chuchu	An imaginary mouse
Mimi	An imaginary rabbit who was a baby

Note. ^a^Study 1 N = 6, Study 2 N = 2.

b Study 1 N = 20, Study 2 N = 15.

After the imaginary companion interview, the children participated in the biological and psychological attributions experiments. To avoid the possibility that children answered the questions in a mode of play or pretense, we considered two points. First, questions were short and did not include predicate-complement sentences [3]. Second, children were given questions with a matter-of-fact intonation and not with make-believe intonation [8].

The procedure of the experiment followed a modified version of Jipson and Gelman’s [24] method. Children were introduced to each agent (a second experimenter, a ball, or an imaginary agent) in random order and then asked a sequence of questions. In the case of the second experimenter, the first experimenter said to children, “She is my friend. I’ll ask you something about her. Can you answer the questions?”). In the case of the ball, the first experimenter told, “This is my ball. I’ll ask you something about it. Can you answer the questions?”). In the case of the imaginary agent, the first experimenter said to the children, “He is my friend, Hikaru. Only I can see him. Can you see him? OK, I’ll ask you something about him. Can you answer the questions?”). The children were then given three biological (“Does X walk?,” “Does X eat?,” “Does X grow?”), three psychological (“Can X feel happy?,” “Can X feel angry?,” “Can X think?”), and three perceptual questions (“Can X see things?,” “Can X hear things?,” “Can X smell things?”) in random order. Children’s responses to each of the property questions were scored for the proportion of “yes” responses given as a function of the question property (biological, psychological, perceptual) and of the items (a human, a ball, an imaginary agent) (range: 0–1.0). A score of “0” meant that children did not attribute each property to the agent, whereas a score of “1” meant that children did attribute each property to the agent. The score of 0.5 was chance level and therefore might mean that children were confused by the questions.

#### Preliminary analyses

We found a significant sex difference between the groups. The IC group was more likely to include girls than the NIC group (Fisher’s exact tests, P<.05), which was consistent with previous research in Western countries, such as USA [5], [32]. Taylor [5] suggested that the gender effects may be due to differences in developmental timetable or type of play. Furthermore, children’s ICs differed as a function of birth order. Among the children with imaginary companions, 19 (73%) were first-borns, while among those without imaginary companions, only eight (33%) were first-borns. This birth order difference was statistically significant (Fisher’s exact tests, P<.05) and also consistent with previous research in Western countries [5]. In terms of birth order effects, it has been suggested that playing with IC may compensate for time to play alone.

We conducted the preliminary analyses to examine whether these factors (sex and birth order) could affect children’s responses to the questions about each agent. The results indicated no significant differences in their response scores for each property and for each item between boys and girls, and between first-borns and other children (P>.05). Therefore, the data for these factors were combined for the subsequent analyses.

### Study 2: An Imagery Stone

#### Participants

A total of 34 children (age range: 37–76 months, mean ± SD: 53.1±11.5) participated in this study. Of these, 14 were boys and 20 were girls. First-born children constituted 53% of the sample. Seventeen children tested into the IC group (age range: 37–76 months, mean ± SD: 54.2±13.8). Among these, four were boys and thirteen girls. The remaining 17 children were placed in the NIC group (age range: 38–67 months, mean ± SD: 52.0±10.6). These included 10 boys and seven girls. No significant differences were observed in the ages between the two groups. Furthermore, no significant differences were found in the verbal age between the IC (mean ± SD: 57.6±14.8) and the NIC (mean ± SD: 51.5±13.3) groups.

#### Procedure

The imaginary companion interview was the same as in Experiment 1.

The procedure was also the same as that in Experiment 1 except that the children were asked about an imaginary stone. The first experimenter said to children, “Here is Ms. Stone. Only I can see her. Can you see Ms. Stone? OK, I’ll ask you something about her. Can you answer the questions?”).

### Study 3: Spontaneous References to Imaginary Items

#### Participants

Participants were the same as those in Experiments 1 and 2.

#### Procedure

We created a situation in which a child might spontaneously talk about the imaginary agent and the imaginary stone. In regard to the imaginary agent, after the initial experiment, the experimenter told the children that the imaginary agent wanted to go to the bathroom and has left the experiment room. Then, the first and second experimenters and children played for several minutes. In regard to the imaginary stone, after the initial experiment, the experimenter told the children that she placed the stone nearby, after which the first and second experimenters and children played.

We observed whether children spontaneously talked about the imaginary items to the experimenters. Specifically, we coded the words which children assigned to the biological, psychological, or perceptual properties of the imaginary agents. We coded words such as “He saw it” “He came back” because in this case, children indeed attributed the perceptual (see) and biological (move) properties to the agent (Table 2). Six children spontaneously mentioned attributes related to the imaginary agent. Two independent coders judged whether the children’s speech included biological, psychological, or perceptual properties. The results of the judges perfectly matched.

## Supporting Information

Movie S1 **A participant who spontaneously referred to an imaginary agent.**
(ZIP)
